# Connexons and pannexons: newcomers in neurophysiology

**DOI:** 10.3389/fncel.2014.00348

**Published:** 2014-11-04

**Authors:** Giselle Cheung, Oana Chever, Nathalie Rouach

**Affiliations:** Neuroglial Interactions in Cerebral Physiopathology, Center for Interdisciplinary Research in Biology, Collège de France, CNRS UMR 7241, INSERM U1050, Labex Memolife, PSL Research UniversityParis, France

**Keywords:** neurons, astrocytes, synapses, plasticity, learning and memory, connexins, pannexins, hemichannels

## Abstract

Connexin hemichannels are single membrane channels which have been traditionally thought to work in pairs to form gap junction channels across two opposing cells. In astrocytes, gap junction channels allow direct intercellular communication and greatly facilitate the transmission of signals. Recently, there has been growing evidence demonstrating that connexin hemichannels, as well as pannexin channels, on their own are open in various conditions. They allow bidirectional flow of ions and signaling molecules and act as release sites for transmitters like ATP and glutamate into the extracellular space. While much attention has focused on the function of connexin hemichannels and pannexons during pathological situations like epilepsy, inflammation, neurodegeneration or ischemia, their potential roles in physiology is often ignored. In order to fully understand the dynamic properties and roles of connexin hemichannels and pannexons in the brain, it is essential to decipher whether they also have some physiological functions and contribute to normal cerebral processes. Here, we present recent studies in the CNS suggesting emerging physiological functions of connexin hemichannels and pannexons in normal neuronal activity and behavior. We also discuss how these pioneer studies pave the way for future research to extend the physiological relevance of connexons and pannexons, and some fundamental issues yet to be addressed.

## Introduction

A typical feature of glia, in particular astrocytes, is to express high levels of connexins (Cxs), which have long been thought to only provide the molecular basis for the formation of gap junction (GJ) channels, mediating the extensive direct glial intercellular communication (Spray et al., 1999; Rozental et al., 2000; Theis et al., 2005; Pannasch and Rouach, 2013). Indeed Cxs, which represent a family of so far ~20 isoforms identified in mice and humans (Willecke et al., 2002), form homomeric or heteromeric hexamers on cell plasma membranes. These structures also called connexons, can align and dock with other connexons provided by neighboring cells to form GJ channels (Sáez et al., 2003). Such GJ channels thus connect the cytoplasm of adjacent cells. They allow direct exchange of a variety of small molecules <1.5 kDa (Loewenstein, 1981), including ions, energy metabolites, neurotransmitters and signaling molecules, coordinating electrical and metabolic activities of connected cells (Dermietzel and Spray, 1993; Pannasch and Rouach, 2013). Importantly, GJ functions have been attributed to various CNS pathologies as both protective and destructive (Rouach et al., 2002; Eugenin et al., 2012). However, over the last years, increasing evidence has emerged showing that astrocytic gap-junctional networks can also modulate physiological activities like synaptic transmission, plasticity and information processing (Lutz et al., 2009; Pannasch et al., 2011; Pannasch and Rouach, 2013; Han et al., 2014).

Nevertheless, besides the classical formation of GJ channels, connexons do exist on their own as single membrane channels, named hemichannels (HCs), which directly connect the cell cytoplasm to the extracellular space. Because HCs are thought to be poorly selective large pore channels permeable to numerous low molecular weight molecules, their opening is commonly viewed as deleterious due to potential loss of cytoplasmic integrity and neurotoxic damage that may be induced by the released factors (Giaume et al., 2013). Such membrane HCs were thus at first presumed to be closed and to serve as a reserve pool of connexons ready to be assembled into GJ channels at junctional plaques. However, this concept has been challenged in the nineties, when Cx HCs were for the first time reported to open in a number of conditions, albeit mostly related to pathological situations, as recently reviewed (Orellana et al., 2012b, 2013; Giaume et al., 2013).

Remarkably another family of proteins, the pannexins (Panxs), was discovered in the early 2000s to be homologous to the invertebrate GJ forming proteins innexins (Baranova et al., 2004). Although Panxs were initially suspected to form GJ channels due to their structural similarities to Cxs, thus far they have actually been shown in native systems to only form large pore membrane channels, similar to Cx HCs. Differential expression and properties of Cx HCs and Panx subtypes in glial cells (Cx43 and Panx1 in astrocytes; Cx43, Cx32, and Panx1 in microglia; Panx1 and possibly Cx29 or Cx32 in oligodendrocytes) and neurons (Cx36 and Panx1) have already been summarized in several comprehensive reviews (Sáez et al., 2003; Thompson and Macvicar, 2008; Orellana et al., 2011c; Giaume et al., 2013). In addition, like Cx HCs, the activation of Panx channels is generally also viewed as detrimental and has accordingly been mostly reported during pathological conditions, as recently extensively reviewed (Bennett et al., 2012; Giaume et al., 2013; Mylvaganam et al., 2014; Orellana et al., 2014; Velasquez and Eugenin, 2014). Interestingly, with these growing findings on HCs properties and roles inside and outside the brain, it was hypothesized that these channels may also have their importance in physiology. Indeed, a few pioneer studies have shown that HCs do open in physiological conditions in the retina (Pearson et al., 2005) or in the inner ear (Anselmi et al., 2008). This has led to the emergence of several recent studies over the last few years focusing on their physiological functions. In particular, Cx43 HCs, and Panx1 channels, among others, have been most commonly investigated in various physiological contexts. Although not much is known about Cx and Panx single membrane channels in the brain during physiological conditions and if they could modulate synaptic transmission and behavior, a few recent studies have explored this possibility. Here, we review these findings on connexons and pannexons focusing on their novel neurophysiological and behavioral roles in the CNS, as well as the experimental approaches used. In addition, future directions and prospects in unraveling the physiological relevance of these channels are also discussed.

## Unique features of connexins and pannexins as single membrane channels

Cx HCs and Panx channels are unique membrane channels due to their large pores with specific conductance properties (Thompson and Macvicar, 2008). This type of channels potentially represents a more dynamic aspect of such transmembrane pores, as their opening to the extracellular space may promote direct regulation of neurotransmission and paracrine signaling. While functions of Cx GJ channels have been extensively investigated and characterized, the properties of Cxs and Panxs as unapposed single membrane channels, or HCs, have only recently been given attention and explored. Over the last years, different experimental approaches have been developed to investigate the properties of HCs. As summarized in Table 1, these include the use of tracers permeable to HCs, quantification of released molecules and electrophysiological recordings of HC currents. Moreover, HC activities can also be manipulated by the addition of mimetic peptides, pharmacological blockers, antibodies or various transgenic animals. These techniques have greatly contributed to the identification of many unique features of Cx HCs and Panx channels.

**Table 1:** **Overview of available techniques and tools to study hemichannels**.

**How to study**	**Technique/Tool**	**Description**	**Useful for**	**Advantages**	**Disadvantages**
HC properties	Dye uptake or efflux	Fluorescence quantification of the uptake or efflux of HC-permeable tracers (e.g., Ethidium bromide, Calcein)	Assessing HC activity based on their permeability to fluorescent tracers	Simple and fast assay	Low temporal resolution
				Assesses HC activity of all cells with open HCs in a preparation	Dye uptake: overestimation of the permeability of HCs due to diffusion of the tracers to neighboring cells via GJ upon uptake
			*In vitro* and *in situ*	Can reveal cell type specific uptake using immunostaining	
				Potential use *in vivo*	
	Efflux of biologically active molecules	Quantification of the release of active molecules (e.g., ATP, glutamate, NAD^+^) via HCs when combined with molecular Cx/Panx knockdown/knockout or pharmacological blockers	Assessing HC activity based on relevant molecules released	Functionally relevant	Low temporal resolution
				Quantitative	Not cell specific
			*In vitro* and *in situ*		
	Electro-physiology	Patch-clamp recordings of HC-mediated ionic currents in whole cell, inside-out or cell-attached configurations	Measuring HC selectivity, kinetics and gating properties	Precise and dynamic measures of biophysical properties of HCs	Invasive
					Not appropriate for *in situ* recordings, due to low space-clamp of cells such as astrocytes
			*In vitro*		
Impact of HCs	Pharmacological blockers	Carbenoxolone, lanthanum, divalent ions, flufenamic acid, alcohols, probenecid	Studying acutely the impact of HC activity	Easy to use	Not cell-specific
			*In vitro*, *in situ* and *in vivo*	Fast action	Not selective for Cxs
	Antibodies	aEL2-186 (Hofer and Dermietzel, 1998), Cx43^E2^ (Orellana et al., 2012b; Riquelme et al., 2013)		Local application and washout possible	presenting high sequence homology
				Antibodies: allow localization of HCs using immunostaining	Potential side effects
	Mimetic peptides	Blockers: Gap26, Gap27, Gap19/TAT-Gap19, L2/TAT-L2, Panx1 and Panx2 mimetic peptides Enhancer: TAT-Cx43CT10 or TAT-Cx43CT9 (favors the opening of Cx43 HCs by preventing their closure at high cytoplasmic Ca^2+^ concentration) (Ponsaerts et al., 2010; De Bock et al., 2012)		Gap19/TAT-Gap19 and TAT-L2: specific blockers for Cx43 HCs but not GJs	Most blocking agents also inhibit GJ functions. This also applies to mimetic peptides when they are used over long periods (Samoilova et al., 2008)
	Small interfering RNAs and Oligo-nucleotides	Genetic downregulation of the expression of Cx43 (Valiunas and Weingart, 2000; Figiel et al., 2007) and Panx1 (Wicki-Stordeur and Swayne, 2013)	Investigating the effect of lowering the expression of HC proteins	High selectivity	Effect dependent on transfection/infection efficiency
			*In vitro*, *in situ* and *in vivo*	Spatial and temporal control of gene knockdown	Off-target effects
	Transgenic mice	**Loss of function**	Studying the impact of chronic alterations in HC activity	Cell specificity using conditional knockout animals	Possible developmental and compensatory effects
		Panx and Cx knockout mice Cx30^T5M/T5M^ mice (defective Cx30 channel pore due to a single point mutation) (Grifa et al., 1999; Essenfelder et al., 2004; Schutz et al., 2010)			
		**Gain of function**	*In vitro*, *in situ* and *in vivo*	Investigation *in vivo*	Irreversible
		hGFAP-CreCx43^+/G138Rfl^ mice (enhanced Cx43 HC activity in GFAP positive cells due to a single point mutation) (Dobrowolski et al., 2008; Torres et al., 2012)		Can be used to test selectivity of Cx and Panx mimetic peptides	Gene alteration also affects GJ and non-channel function of Cx and Panxs

aEL2-186, antibody against position 186–206 on the extracellular loop of Cx43; Cx, Connexin; Cx43CT9 and 10, peptides targeting the last 9 or 10 amino acids of the Cx43 C terminus; Cx43^E2^, antibody against the second extracellular loop (E2 domain) of Cx43; fl, floxed; G138R, substitution of a glycine by an arginine at position 138 of Cx43; GFAP, glial fibrillary acidic protein; GJ, gap junction; HC, hemichannel; Panx, Pannexin; TAT-, peptide variant with enhanced plasma membrane permeability; T5M, substitution of a threonine by methionine at position 5 of Cx30.

### Connexin hemichannels

The first studies to show evidence for functional HCs was on rat lens fiber Cx46 expressed in xenopus oocytes (Paul et al., 1991; Ebihara and Steiner, 1993). In particular, the authors have identified non-junctional voltage dependent currents in Cx46 expressing oocytes, which they have later confirmed in lens fiber (Ebihara et al., 2011). Functional Cx30, Cx46 and Cx50 HCs have also been detected using HeLa cell expression system, but were found to be closed during physiological conditions (Valiunas and Weingart, 2000). Indeed, extracellular binding of Ca^2+^, intracellular phosphorylation and strong voltage-dependence are hindering the opening of Cx HCs near resting membrane potentials. More subsequent studies have observed similar HC properties of Cx30.2 and Cx31.9 (Bukauskas et al., 2006), Cx43 (Contreras et al., 2003; Kang et al., 2008), and Cx26 (Gonzalez et al., 2006), although human Cx26 HCs have been shown in the inner ear to open in physiological conditions, where they sustain long range intercellular Ca^2+^ signals (Anselmi et al., 2008). Noteworthy, a physiologically low Ca^2+^ level inside cochlear endolymph (Bosher and Warren, 1978) is likely the reason why Cx26 HCs are open and participate in physiological signaling in the inner ear. Remarkably, HCs properties differ according to the Cx composition, which confers distinctive permeability and biophysical properties, including open probability, conductance and selectivity (Giaume et al., 2013). In addition, Cx HCs are strongly regulated, and some triggers for their opening include transmembrane voltage, changes in Ca^2+^ and K^+^ concentrations, ATP or post-transcriptional modifications of the channels, which have been extensively reviewed (Sáez et al., 2005; Macvicar and Thompson, 2010; Giaume et al., 2013; Orellana et al., 2013; Wang et al., 2013a). Finally, these HCs provide a direct means for signaling molecules like ATP and glutamate to travel between intra- and extracellular space (Giaume et al., 2013). In particular, the release of extracellular messengers like ATP via Cx HCs are of significant importance and has been shown to contribute to astroglial Ca^2+^ waves (Leybaert and Sanderson, 2012). Since their discoveries, many other signaling molecules like NAD^+^, glutamate, glutathione and prostaglandin E2 have been found to be released via Cx HCs (Wang et al., 2013a), suggesting multiple dynamic functions of Cx HCs.

### Pannexin channels

In 2004, a new type of GJ proteins homologous to the invertebrate innexins family was cloned (Baranova et al., 2004). Three members of this family, the Pannexins (Panxs) have been identified, with Panx1 expressed ubiquitously, Panx2 specifically in the brain, and Panx3 in osteoblasts and synovial fibroblasts (Baranova et al., 2004). In addition, Panx1 are found to be more abundant during early neuronal development and Panx2 later (Penuela et al., 2007). Interestingly, with little sequence homology, the Panxs share very similar topology and structure with the Cxs, displaying four transmembrane domains, two extracellular- and one intracellular- loop, as well as intracellular N- and C-termini (D'hondt et al., 2009). However, in contrast to Cxs, Panxs normally act in native systems as single membrane large pore channels rather than GJs (Penuela et al., 2007; Sosinsky et al., 2011). In fact, the only evidence of Panx GJ formation was demonstrated in overexpression systems in specific cell lines and showed distinct properties from Cx GJ channels (Bruzzone et al., 2003; Lai et al., 2007; Sahu et al., 2014). Moreover, like Cx HCs, Panx channels can release numerous signaling molecules such as ATP or glutamate (Ye et al., 2003; Iglesias et al., 2009; Giaume et al., 2013). Remarkably, a unique property of Panxs is that they are glycosylated extracellularly at an arginine residue (Boassa et al., 2008). This glycosylation process has been proposed to be the limiting factor preventing Panx channels on adjacent cells from forming GJ channels. Thus, rare gap-junctional formation was only observed when Panx1 was overexpressed to a level exceeding glycosylation capacity (Bruzzone et al., 2003; Lai et al., 2007; Sahu et al., 2014). Concerning the biophysical properties of Panx channels, they are still elusive and controversial, most likely due to the recent discovery of Panxs compared to Cx HCs. Indeed, for instance unitary conductance of Panx1 channels has been reported to range from ~70 to 550 pS, which may reflect different recording conditions, cell type properties or sub-conductance states (Locovei et al., 2006; Kienitz et al., 2011; Ma et al., 2012). While the expression of Panx1 overlaps with that of Cxs mediating extensive GJ coupling in cellular networks (Ray et al., 2005), it was found to be distinctively regulated as compared to the Cxs (Penuela et al., 2007), implying unique functional significance of Panx channels. For instance, while many reports have demonstrated sensitivity of Cx HCs to extracellular Ca^2+^ ([Ca^2+^]_e_), which is commonly thought to keep HCs in a closed state at physiological [Ca^2+^]_e_ (Ebihara et al., 2003), Panxs appear to be insensitive to this (Bruzzone et al., 2005). In addition, Panx1 channels do not show the strong voltage-dependence typical of Cx HCs (Bruzzone et al., 2003). Thus, this type of insensitivity suggests that Panx channel activity does not depend on neuronal activity and that Panx channels might be open during basal physiological conditions.

## Emerging roles of connexon and pannexon channels in neurophysiology and behavior

Cx HCs and Panxs as single membrane channels have undoubtedly many roles in CNS pathologies involving inflammation (Orellana et al., 2011a; Bennett et al., 2012; Makarenkova and Shestopalov, 2014), ischemia (Contreras et al., 2004; Bargiotas et al., 2011), epilepsy (Thompson et al., 2008) and neurodegeneration (Orellana et al., 2011b, 2012b). For a long time, it was believed that Cx HCs and Panx channels with large channel conductance would remain closed in normal conditions in order to maintain cellular integrity and to prevent unregulated transmitter release into the extracellular space. It is indeed conceivable that, like many membrane channels, these Cx HCs and Panxs serve as a direct and effective way to relieve cells from any homeostatic imbalances found in many pathological situations (Bennett et al., 2012; Giaume et al., 2013). Given that specific HCs are found to be active outside the brain in physiological settings (Anselmi et al., 2008), and that Panx channels have unique properties favoring channel opening under the same conditions, a few pioneer groups began to postulate that these channels might also have some relevance in basal neuronal processes. Here, we review the few recent studies examining roles of Cx and Panx single membranes large pore channels on physiological functions ranging from cell division to learning and memory.

### Development

During CNS development including embryonic and adult neurogenesis, purinergic signaling is believed to have crucial contributions (Zimmermann, 2011). Indeed, ATP receptor activation was found to play a role in proliferation and DNA synthesis in astrocytes (Neary and Zhu, 1994) and neural stem cells (Ryu et al., 2003). The fact that both Cx43 HCs and Panx1 channels are permeable to ATP makes them candidates for purinergic signaling during development. The involvement of Cx43 HCs in these fundamental cellular processes was first explored in embryonic retina. As a result, they were found to play a role in cell division and proliferation (Pearson et al., 2005). The authors have demonstrated that upon spontaneous Ca^2+^ increase in a “trigger cell” in the retinal pigment epithelium, ATP is released via Cx43 HCs into the extracellular space adjacent to retinal progenitor cells during development, promoting mitosis and proliferation. In addition, the released ATP also triggers the spread of Ca^2+^ waves through neighboring retinal pigment epithelial cells. Both of these mechanisms involve P2 receptor activation. A short application (30 min) of the mimetic peptide Gap26, which specifically blocks Cx43 HCs without affecting GJ functions, was used to test the involvement of Cx43 HCs, whereas HC activity in retinal pigment epithelial cells was measured by Alexa 488 dye efflux assay. In a more recent study, functional Panx1 channels were identified in postnatal neural stem and progenitor cells both *in vitro* and *in vivo* (Wicki-Stordeur et al., 2012). Panx1 channel specific effect was investigated using Panx1 siRNA knockdown *in vitro* and/or probenecid, a blocker of Panx1 channels without affecting Cx HCs. These results were found to positively regulate proliferation of these cell types via ATP release and subsequent P2 receptor activation. The same group later demonstrated that Panx1 also plays a role in cell migration and neurite extension through its interaction with actin cytoskeleton (Wicki-Stordeur and Swayne, 2013). These results are interesting, since adult neurogenesis plays important roles in both physiological brain development as well as brain repair during pathology and diseases (Berg et al., 2013). Although astrocytic Cx43 and Cx30 have been shown to differentially regulate adult neurogenesis in knockout mice (Liebmann et al., 2013), it was not clear whether it was via their GJ or HC properties. The potential involvement of Cx43 HCs in embryonic retinal development and Panx1 channels in adult neurogenesis is encouraging and may offer important insights into other possible non-synaptic physiological functions of single membrane channels yet to be discovered.

### Glucose sensing and signal transduction

Apart from promoting cell division and proliferation, the activities of Cx43 HCs and Panx1 channels have also recently been proposed to be modulated by changes in extracellular glucose concentrations. Neuronal glucose-sensing is an important physiological process in the hypothalamus, in which action potentials are driven by changes in extracellular glucose concentration. This has been shown to largely contribute to feeding and satiety behavior, sleep-wake cycles, and energy expenditure (Levin et al., 2004; Burdakov et al., 2005). In astrocytes, glucose uptake via Cx43 HCs has already been demonstrated under inflammatory conditions (Retamal et al., 2007). However, whether this is also true in physiology remains unclear. Recently, the role of glial Cx43 HCs in glucose-sensing was explored using cultured tanycytes, which are specialized glial cells in the hypothalamus (Orellana et al., 2012a). The authors showed that, upon a rise in extracellular glucose concentration, glucose transporters (GLUTs) and to a lesser extent Cx43 HCs, allow diffusion of glucose into tanycytes, where it leads to increase in ATP. Subsequently, ATP is released via Cx43 HCs, which then stimulates P2Y receptors locally leading to the rise of [Ca^2+^]_i_. Both ATP release and [Ca^2+^]_i_ responses were inhibited by Cx43 HC blocking agents like La^3+^, Gap26, and Cx43^E2^ (an antibody against the second extracellular loop of Cx43). The opening of Cx43 HCs was found to be promoted by the closing of K_ATP_ channels. Interestingly, this serie of events does not require extracellular Ca^2+^. Using ethidium bromide uptake assay, electrophysiology, and surface biotinylation, they have demonstrated that the open probability of Cx43 HCs, rather than their number, was enhanced by glucose. Although it was recently demonstrated that Cx30 but not Cx43 HCs expressed on oocytes are permeable to glucose (Hansen et al., 2014), these results have revealed a new role of Cx43 HCs in physiological situation, namely to sense and metabolize extracellular glucose. Further studies on how this directly affects glucose metabolism in the hypothalamus, as well as behavioral modifications *in vivo* would largely complement these data and draw physiological relevance. Since tanycytes express a majority of Cx43 rather than Panxs, it is not surprising that Panxs were found not to be involved in this process. However, it would be interesting to explore whether astrocytic Panxs participate in similar signaling pathways. In fact, the involvement of Panxs in metabolic autocrine regulation has been previously demonstrated (Kawamura et al., 2010). The authors showed that non-pathological changes in extracellular glucose concentration induce a purinergic autoregulation in hippocampal CA3 neurons. This was found to be mediated by the opening of Panx1 channels on neurons, but not astrocytes. Although this study was designed to determine the effect of the anti-epileptic ketogenic diet (high fat and low carbohydrates) on increasing seizure threshold, it gave important insights into how Panx1 channels could be involved in regulating physiological metabolic perturbations.

### Neuronal excitability and synaptic transmission

**Figure 1:**
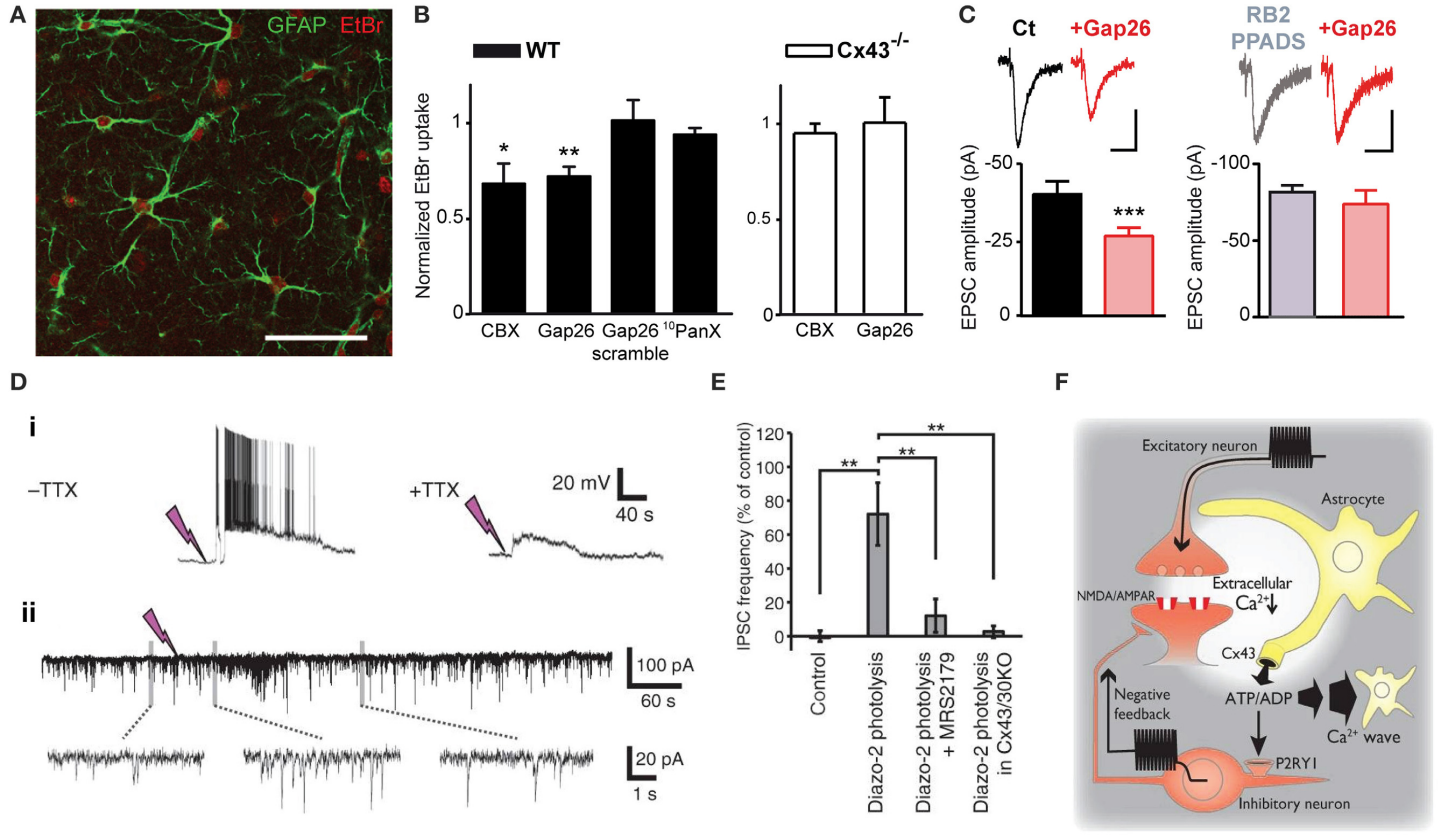
**Astrocytic Cx43 HCs modulate synaptic transmission in hippocampal slices. (A–C)** Basal astroglial Cx43 HC activity enhances excitatory synaptic transmission via ATP signaling. **(A)** Representative image showing ethidium bromide uptake (EtBr; red) in astrocytes (immunostained for GFAP; green) of the stratum radiatum in an acute hippocampal slice. Scale bar, 50 μm. **(B)** Bar graphs showing astrocytic EtBr uptake in brain slices obtained from wild-type (WT) and astroglial conditional Cx43 KO (Cx43^−/−^) mice normalized to control (untreated) conditions. Uptake was significantly deceased in WT slices treated with carbenoxolone (CBX, 200 μM) and Gap26 (100 μM), but not Gap26 scramble (100 μM) and ^10^panx (400 μM) peptides. In Cx43^−/−^ slices, however, both CBX and Gap26 had no significant effect. **(C)** Bar graph on the left showing a decrease in amplitude of evoked EPSC recorded in CA1 pyramidal neurons during Gap26 application (red) as compared to before (Ct, black). Bar graph on the right showing that pretreatment with ATP P2 receptor antagonists (RB2 + PPADS, gray) occludes the effect of Gap26 (red). Sample traces of corresponding evoked EPSCs are shown above. Scale bar: 20 pA, 20 ms (left); 40 pA, 40 ms (right). **(D–F)** Cx43 HCs in astrocytes promote feedback inhibitory transmission by releasing ATP. **(D)** Representative recordings showing that photolysis of diazo-2, represented by lightning bolts, (i) evokes depolarization and bursting in interneurons, with the depolarization persisting with 1 μM TTX, and (ii) transiently increases the frequency of spontaneous inhibitory postsynaptic currents (IPSCs) in CA1 pyramidal neurons. **(E)** Bar graph indicating increased IPSC frequency in pyramidal neurons after diazo-2 photolysis compared to control condition. This effect was blocked by the P2Y1 receptor antagonist MRS2179 (50 μM) or in brain slices prepared from Cx43/Cx30KO mice. **(F)** Schematic diagram illustrating a proposed negative feedback mechanism during excitatory transmission. During glutamatergic signaling, Ca^2+^ influx into neurons results in a localized decrease in [Ca^2+^]_e_, which in turn opens Cx43 HCs on astrocytes through which ATP is released. ATP can either trigger slowly propagating astrocytic Ca^2+^ waves or, when degraded to ADP, depolarize and increase firing in interneurons via P2Y1 receptors, thereby enhancing inhibitory transmission. ^*^p < 0.05; ^**^p < 0.01; ^***^p < 0.001. Adapted, with permission, from Torres et al. (2012) **(D–F)**.

Astrocytes express a large repertoire of ions channels, neurotransmitter receptors and transporters, allowing them to sense and modulate neuronal activity through multiple mechanisms. These include in part intracellular calcium signaling, morphological changes or gliotransmission, in which neuroactive substances such as glutamate or ATP are released from astrocytes and act on neurons. The opening of Cx HCs and Panx channels during physiological conditions suggests that they represent a release pathway of gliotransmitters and ions, which can regulate neuronal excitability and basal synaptic transmission. Here, we first present findings related to ATP release via astroglial Cx43 HCs and synaptic transmission. In particular, among several recent studies demonstrating physiological relevance of Cx43 HCs, we have very recently shown that astroglial Cx43 HCs are not only open in acute hippocampal slices under physiological conditions, but can also modulate basal excitatory synaptic transmission through ATP signaling (Chever et al., 2014). Specifically, we reported that ethidium bromide uptake into astrocytes under basal conditions was decreased in the presence of the HC blocker carbenoxolone (CBX) or the Cx43 HC mimetic blocking peptide Gap26. These blocking agents had no effect on ethidium bromide uptake in brain slices prepared from Cx43^fl/fl^ hGFAP-Cre mice (Cx43^−/−^). These strongly suggested that functional astroglial Cx43 HCs are present under physiological conditions (Figures 1A,B). Interestingly, excitatory postsynaptic currents recorded in CA1 pyramidal neurons and ATP release from hippocampal slices detected using a luciferin-luciferase luminescence assay were also concurrently decreased in the presence of Gap26. Furthermore, pretreatment with ATP P2X and P2Y receptor antagonists abolished the inhibitory effect of Gap26 on excitatory synaptic activity, suggesting the involvement of P2 receptors (Figure 1C). Our results thus suggest a novel physiological pathway involving functional astroglial Cx43 HCs and ATP release in tuning excitatory synaptic transmission in the hippocampus.

The involvement of HCs in promoting astrocytic Ca^2+^ waves by ATP release (Cotrina et al., 1998) suggested another possible means for HCs to contribute to physiological cell-cell interaction and propagation of signals. In fact, this has already been demonstrated in the inner ear. Both Cx26 and Cx30 were found to play a role in propagating long-range intercellular Ca^2+^ signaling in cochlear organotypic cultures (Anselmi et al., 2008). These Cxs contributed by HC-mediated ATP release as well as gap-junctional diffusion of Ca^2+^-mobilizing second messengers. A recent study (Torres et al., 2012) has taken a different approach to study HC function by directly decreasing [Ca^2+^]_e_ (by 0.5 mM), to a range thought to occur during neuronal network activity (Massimini and Amzica, 2001; Amzica et al., 2002). Since low [Ca^2+^]_e_ has been found to open Cx HCs (Sáez et al., 2005), the effect of lowering [Ca^2+^]_e_ on synaptic transmission and the involvement of HCs in this process was investigated. Specifically, [Ca^2+^]_e_ in acute hippocampal slices was decreased using several methods including uncaging Ca^2+^ chelator (diazo2) or glutamate (MNI-glutamate) extracellularly. In an attempt to draw physiological relevance, high frequency stimulation-induced decrease in [Ca^2+^]_e_ was also assessed. With these manipulations to decrease [Ca^2+^]_e_, they detected an increase in extracellular ATP concentration, which triggered a slow astrocytic Ca^2+^ wave. Such ATP release was proposed to be mediated by astroglial Cx43 HCs, using a combination of molecular and pharmacological tools. Cx30^−/−^ Cx43^fl/fl^ hGFAP-Cre mice, with conditional deletion of Cx43 in astrocytes and total deletion of Cx30 (Wallraff et al., 2006), were used to avoid compensatory up-regulation of Cx30 (Theis et al., 2003). Astroglial Ca^2+^ waves were abolished in these mice. In comparison, Cx30^−/−^ Cx43^fl/fl^ mice (Cx30KO) were used to show that no alteration in astrocytic Ca^2+^ waves was observed with functional Cx43. In addition, transgenic mice with an astrocyte-targeted point mutation of Cx43 (glycine 138 substituted with arginine) were also used. In such mice, thought to display an increased number of open Cx43 HCs, but also deficient gap junctional coupling (Dobrowolski et al., 2008), astroglial Ca^2+^ waves were enhanced. Finally, CBX, a pharmacological inhibitor of both Cx HCs and GJ channels was found to abolish the evoked astroglial slow Ca^2+^ waves. In these experiments, which exclude a contribution of Cx30 in the evoked astroglial slow Ca^2+^ waves, whether Cx43 GJ channels also contribute to astroglial Ca^2+^ signaling is still an open question. Interestingly, the authors also showed that ATP released by Cx43 HCs subsequently activated P2Y1 receptors on inhibitory interneurons, which potentiated their excitability and thereby inhibitory synaptic transmission (Figures 1D,E). Thus, in response to intense neuronal activity where [Ca^2+^]_e_ is significantly decreased, astrocytic Cx43 HCs were proposed to play a complex role in a negative feedback mechanism by initiating and propagating inhibition to tone down strong excitatory transmission and hypexcitability of neuronal networks (Figure 1F). This study suggests an interesting link between HC-mediated astrocytic Ca^2+^ waves and inhibitory synaptic transmission in response to intense neuronal activity. However, it is not clear whether all the experimental protocols used in this study generated a decrease in [Ca^2+^]_e_ that is within a physiologically relevant range. Additional studies are therefore warranted to demonstrate that the regulation of neuronal activity by astroglial Cx43 HCs also applies to physiological conditions. In addition, it is unclear whether the decrease in [Ca^2+^]_e_ used in this study induces significant Cx HC opening in astrocytes. Indeed, although slight decrease in [Ca^2+^]_e_, from 1.8 to 1.6 mM, has been found to open Cx HCs in cell lines (Quist et al., 2000), in cultured astrocytes [Ca^2+^]_e_ below 1 mM is necessary to significantly open HCs, as assessed by HC-mediated glutamate release (Ye et al., 2003). Finally, it still remains unknown whether ATP activated P2Y1 receptors in other cells than interneurons, such as astrocytes, where they can contribute to glutamatergic gliotransmission (Jourdain et al., 2007; Pascual et al., 2012).

**Figure 2:**
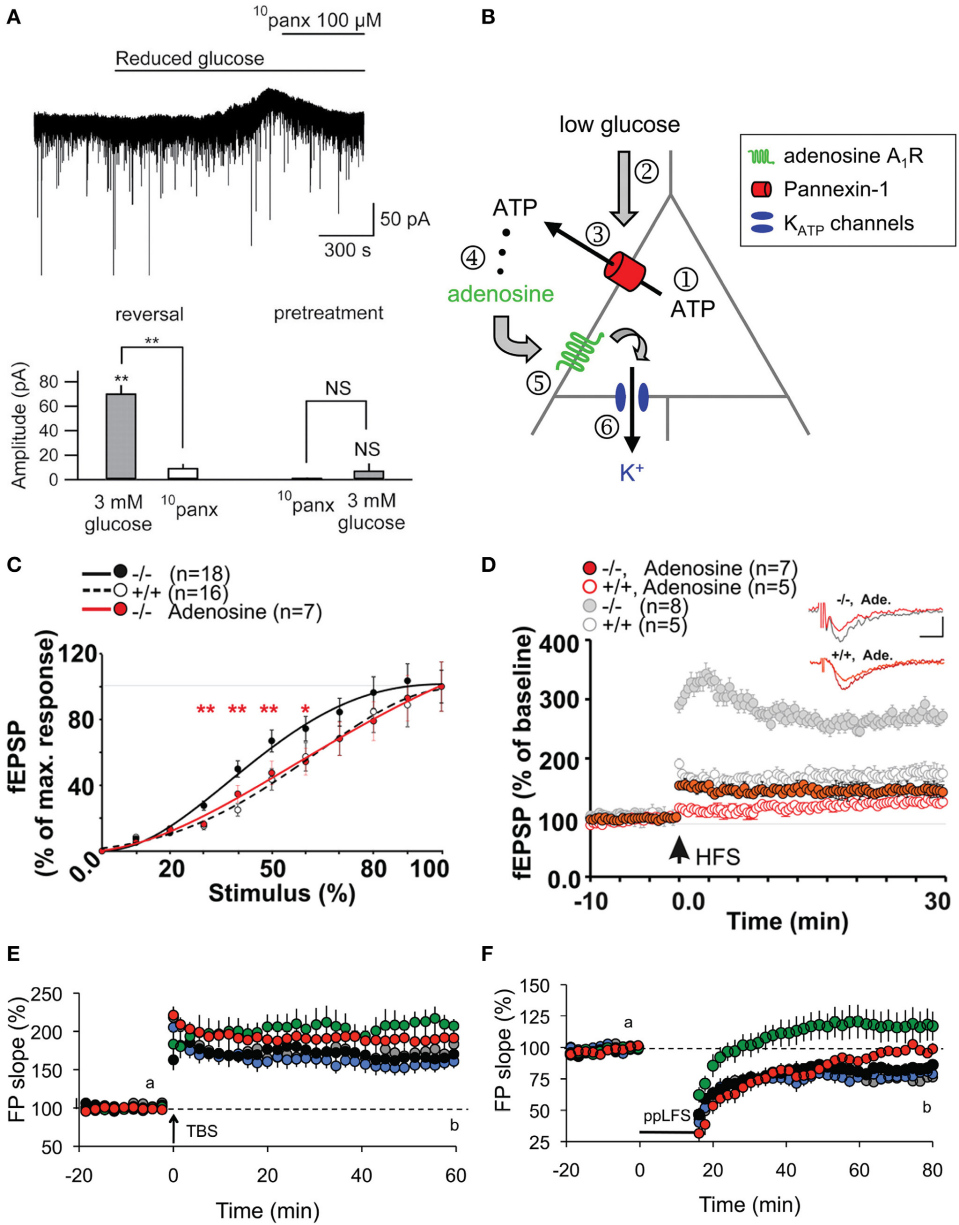
**Panx1 channels modulate neuronal excitability, synaptic transmission and plasticity in hippocampal slices. (A,B)** Metabolic autocrine regulation of neuronal activity via Panx1 channels and adenosine. **(A)** Sample trace showing increased outward current upon reduced extracellular glucose (from 11 to 3 mM) and subsequent reversal to baseline with ^10^panx application (100 μM) in CA3 pyramidal neurons. Bar graphs showing the reversal of reduced glucose-induced outward current amplitude with ^10^panx (left), and that pretreatment with ^10^panx prevented reduced glucose-induced outward current. ^**^p < 0.01. **(B)** Schematic showing a proposed model of purinergic autocrine regulation in CA3 pyramidal neurons. When [ATP]_i_ is sufficient (1), low [Glucose]_e_ (2) induces ATP release from Panx1 channels on neurons (3). ATP is then dephosphorylated to adenosine (4) which activates adenosice A_1_R rceptors (5). K_ATP_ channels are then opened leading to a decrease in neuronal excitability. **(C–F)** Panx1 regulates synaptic transmission, LTP and LTD. **(C)** Input-Output curves showing increased synaptic transmission in Panx1^−/−^ (black line) compared to control Panx1^+/+^ (dashed line) mice. Such effect was abolished in Panx1^−/−^ slices treated with 3 μM adenosine (red line). ^*^p < 0.01; ^**^p < 0.001. **(D)** LTP evoked by high frequency stimulation (four trains of 10 shocks at 100 Hz every 1 s; HFS) is enhanced in Panx1^−/−^ (filled gray) compared to control Panx1^+/+^ (open gray) mice. Adenosine treatment in Panx1^−/−^ slices (filled red) restores LTP levels to that of untreated control mice. Figure insets illustrate responses before and 30 min post HFS. Scale bar: 0.5 mV, 10 ms. **(E)** LTP induced by the delivery of theta burst stimulation protocol (TBS) is increased in adult Panx1^−/−^ (green) compared to Panx1^+/+^ (black) mice, whereas no difference was observed between young mice (+/+, gray; −/−, blue). In the presence of 100 μM probenecid (Panx1 channel blocker; red), only transient LTP was enhanced. **(F)** Similarly, LTD induced by paired-pulse low frequency stimulation protocol (1Hz for 15 min; PP-LFS) are impaired in adult Panx1^−/−^ (green) compared to Panx1^+/+^ (black) mice, whereas no difference was observed between young equivalent (+/+, gray; −/−, blue). In the presence of 100 μM probenecid, only a transient LTD was observed. Adapted, with permission, from Kawamura et al. (2010) **(A,B)**, Prochnow et al. (2012) **(C,D)** and Ardiles et al. (2014) **(E,F)**.

ATP-mediated modulations of neurotransmission are not only restricted to astroglial Cx43 HCs. Indeed, an interesting link between Panx channels, which also release ATP, and basal neuronal excitability was reported (Kawamura et al., 2010). Using whole-cell patch clamp recordings, it was demonstrated that changes in extracellular glucose concentration from 11 to 3 mM opened Panx1 channels on rat hippocampal CA3 pyramidal neurons through which ATP is released. Its metabolite adenosine then activates adenosine A1 receptors, leading to the opening of ATP-sensitive K^+^ channels. As a result of this autocrine regulation, neuronal hyperpolarization occurs, downregulating neuronal activity. The involvement of Panx1 channels was confirmed using CBX, octanol and the selective peptide blocker ^10^panx (Figures 2A,B). Of note, although decreasing glucose concentration to 3 mM was confirmed to be non-pathological using extracellular recordings, an initial concentration of 11 mM is almost four-fold higher than extracellular glucose concentration in brain tissues *in vivo* (Shram et al., 1997). Whether this initial hyperglycemic condition contributed to the Panx1-mediated regulation of neuronal activity thus remains to be clarified.

Following this report, two other studies have demonstrated using Panx1^−/−^ mice and/or pharmacological blockade of Panx1 channels that Panx1 also plays an important role in synaptic transmission. Prochnow and colleagues observed a significant increase in synaptic transmission in adult Panx1^−/−^ mice by measuring the input-output curves at the hippocampal Schaffer-collateral CA1 synapse (Prochnow et al., 2012). Interestingly, this effect was abolished in the presence of adenosine, suggesting that involvement of ATP release via Panx1 channels might be involved (Figure 2C). Similar experiments were later performed by another group (Ardiles et al., 2014). Importantly, this later study showed that the enhanced synaptic transmission in Panx1^−/−^ mice was only observed in adult (9–12 month-old) but not in young (1 month-old) mice. They also saw a similar enhancement in synaptic transmission by treating adult brain slices with a Panx1 channel blocker, probenecid, further confirming their observations. This effect was not related to change in presynaptic release probability, as assessed by paired pulse facilitation. The authors however postulated that Panx1 deletion could reduce ATP release and thus extracellular adenosine levels, leading to an increase in glutamate release, as previously proposed (Prochnow et al., 2012). To indirectly support this hypothesis, they experimentally increased synaptic glutamate levels using a glutamate transporter blocker (TBOA), and observed a larger increase in fEPSP in adult Panx1^−/−^ and probenecid-treated control than in untreated control. However, direct evidence of pre- and/or postsynaptic effects is still lacking. It would also be interesting to determine whether the effects observed are mediated by neuronal and/or glial Panx1 channels.

### Synaptic plasticity, learning, and memory

**Figure 3:**
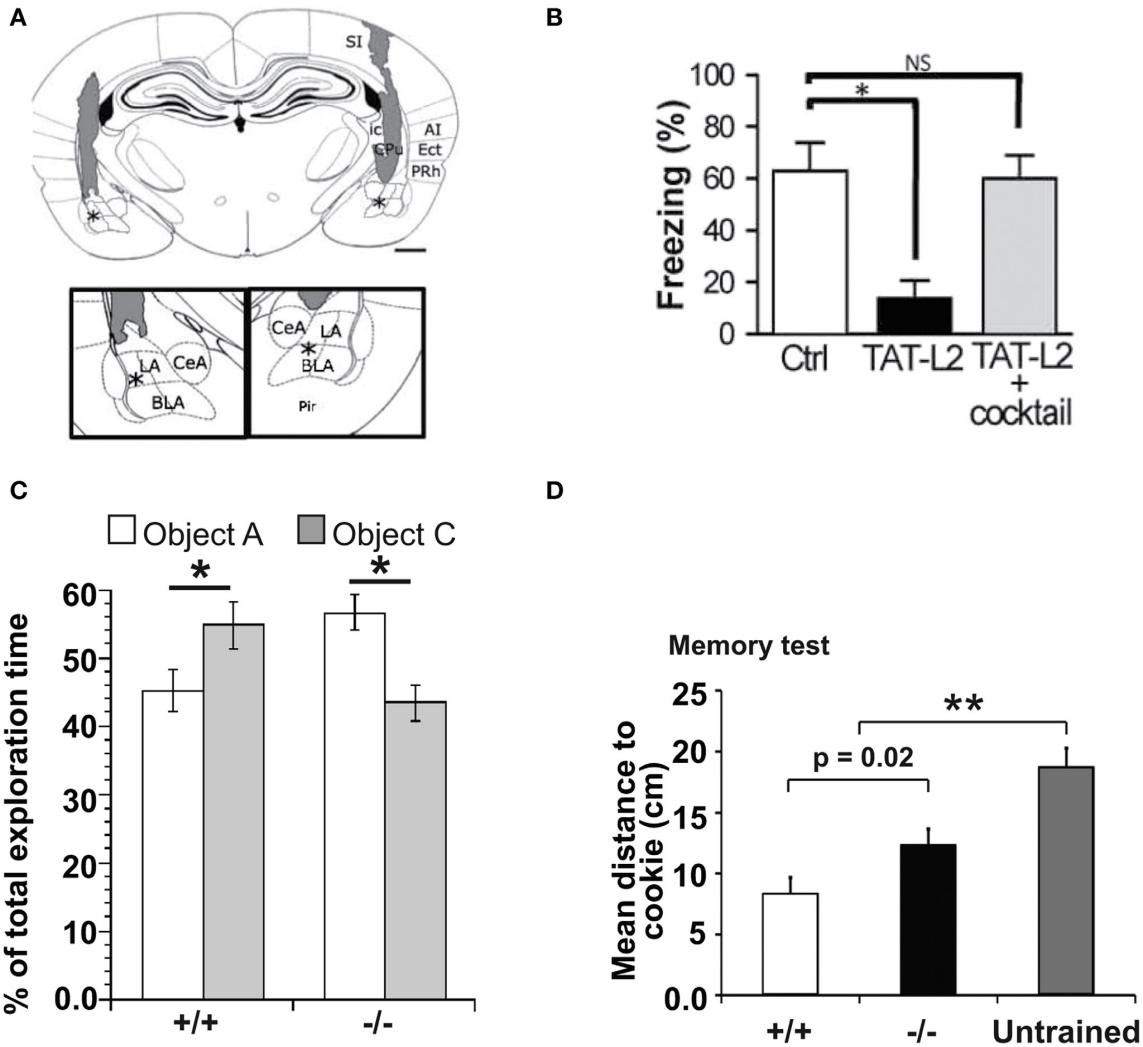
**Cx HCs and Panx1 channels have significant roles in learning and memory. (A,B)** Cx43 HC function is required in fear conditioning memory consolidation. **(A)** Diagram showing site of microinfusions of the TAT-Cx43L2, a selective Cx43 HC blocker, into the basolateral amydala (BLA). Asterisks indicate the tips of injection cannula (shaded regions). Enlargement is shown in insets. LA, lateral amygdala; CeA, central amygdala; SI, somatosensory primary; PRh, perirhinal; Ect, ectorhinal; Pir, piriform; and AI, auditory primary cortices; CPu, caudoputamen; ic, internal capsula. Scale bar: 1 mm. **(B)** Fear conditioning memory was tested by first training rats to associate a tone with a foot shock. Their memory of this association was then assessed by how long they remain immobile (freeze) in response to the same tone alone 24 h later. TAT-Cx43L2 (10 nM) microinfusion prior to training impaired fear conditioning memory (decreased in freezing time) compared to control. Such effect was rescued by co-microinfusion of a mixture of gliotransmitters including D-serine, glutamate, glutamine, glycine, ATP, and lactate (cocktail) rescued such effect. **(C,D)** Panx1 deletion leads to dysfunctions in learning and memory. **(C)** To assess object recognition, mice were allowed to explore two novel objects (A and B) for 5 min. 1 h later, they were allowed to explore the familiar object A together with a novel object C. Panx1^−/−^ mice spent more time on object A than C compared to Panx1^+/+^ mice which did the opposite, indicating a deficit in object recognition. **(D)** Another memory test was carried out where mice were trained to remember locations of hidden cookies which were later removed. During the test, Panx1^−/−^ mice explored locations further from the original locations of cookies compared to the Panx1^+/+^ mice but not as far as the untrained mice, indicating an impairment but not abolishment in memory. ^*^p < 0.05; ^**^p < 0.001. Adapted, with permission, from Stehberg et al. (2012) **(A,B)** and Prochnow et al. (2012) **(C,D)**.

With these growing reports suggesting important roles of Cx HCs and Panx channels in neuronal excitability and synaptic transmission, some groups became interested in studying their functional significance in terms of synaptic plasticity as well as learning and memory. It is indeed believed that astrocytes have many essential roles in synaptic functions, including synaptic plasticity and information processing (Perea et al., 2009; Dallerac et al., 2013; Pannasch and Rouach, 2013). Whether this could be conducted via astroglial HC activity was first investigated in 2012. In particular, a possible role of HCs in learning and memory was reported (Stehberg et al., 2012). The authors selectively blocked Cx43 HCs using TAT-Cx43L2. This peptide was designed to interfere with the intracellular loop/tail interactions of Cx43 necessary for its HC but not gap-junctional activities (Ponsaerts et al., 2010). After microinfusion of TAT-Cx43L2 into the rat basolateral amygdala, an area of the brain associated with emotional memory (Ledoux, 2007), amnesia for auditory fear conditioning was observed (Figures 3A,B). This effect was found to be specific to memory consolidation, as the blocker applied 6 h after learning did not lead to memory deficits. Their results were corroborated using Gap27, a peptide commonly used to block Cx43 HC activity extracellularly, further suggesting specific contributions from Cx43 HCs. In order to investigate the molecular mechanisms involved, a cocktail of gliotransmitters containing glutamate, glutamine, lactate, D-Serine, glycine, and ATP was co-infused with TAT-Cx43L2. As a result, a rescue of the memory defect was observed (Figure 3B). Since they showed that TAT-Cx43L2 had no effect on ATP and glutamate release in neuronal cultures, and that Cx43 was found on astrocytes but not neurons, the authors concluded that the effect was specific for astroglial gliotransmitter release. This pioneer *in vivo* study has suggested an important contribution of gliotransmitter release via Cx43 HCs, presumed to be of astrocytic origin, in memory consolidation. However, it still remains elusive which gliotransmitters are involved and their downstream targets. Furthermore, direct release of such gliotransmitters via Cx43 HCs is yet to be demonstrated, as there are also other possible mechanisms like vesicular exocytosis (Parpura et al., 2004), Panxs (Iglesias et al., 2009), P2X7 channels (Suadicani et al., 2006), Bestrophin 1 (Han et al., 2013), and volume-regulated anion channels (Kimelberg, 2004), which could be downstream effects to Cx43 HC activation.

Shortly following the study by Stehberg and colleagues, another report linking HC activities to learning and memory was revealed. This time, the focus was on Panx channels and their well-characterized role in purinergic signaling. Since Panx1 is strongly expressed in postsynaptic terminals (Zoidl et al., 2007) and presumably also in astrocytes (Iglesias et al., 2009), this study explored the contributions of Panx1 to synaptic plasticity, which in turn may influence learning. Prochnow and colleagues have observed that in Panx1^−/−^ hippocampus, neurons have increased synaptic transmission and enhanced long-term potentiation (LTP) triggered by high frequency stimulation (Prochnow et al., 2012), as shown in Figure 2D. Interestingly, these changes in neuronal properties could be rescued by supplying extracellular adenosine, a metabolite of ATP, as well as blockage of postsynaptic NMDARs, indicating that this process involves Panx1-mediated ATP release and an inhibition of glutamatergic excitatory responses. They have also found that only in adult mice, an upregulation of mGluR4 accompanies chronic deletion of Panx1, which accounted for the enhanced persistent LTP observed. The authors suggested that this might be an adaptive compensatory response to the enhanced neuronal transmission. Consequently, these modifications in Panx1^−/−^ mice led to behavioral defects like altered sensory motor gating capabilities, assessed using pre-pulse inhibition of the acoustic startle response, which may reflect stress and anxiety. Cognitively, novel object recognition (Figure 3C) and spatial learning involving the location of a treat were also impaired (Figure 3D). These deficits were expected, since it has been shown that saturation of LTP is related to impaired learning (Moser et al., 1998). Indeed, this study demonstrated that ATP released through Panx1 channels participates in maintaining synaptic strength and plasticity in hippocampal CA1 neurons, and in turn plays a role in learning and memory. Furthermore, the authors suggested that Panx1 mediates a feedback response through presynaptic activation of adenosine A1 receptors and inhibition of glutamate release, which is essential in cognitive functioning. However, this study did not distinguish between neuronal and glial Panx1 channels. Given that astrocytes are thought to also express ATP-releasing Panx1 channels (Iglesias et al., 2009) and that they have been implicated in the modulation of neuronal activities (Dallerac et al., 2013), it is of interest to determine specific astrocytic contributions in these behavioral and cognitive processes, possibly with the use of conditional knockouts.

Interestingly, very recently, another study showed that not only LTP but also long-term depression (LTD) is altered in Panx1^−/−^ mice (Ardiles et al., 2014). Specifically, they observed that LTD was impaired in either Panx1^−/−^ hippocampus or during pharmacological blockage of Panx1 channels. Moreover, they have also demonstrated that both the enhancement in LTP and impairment in LTD were only observed in adult but not in young Panx1^−/−^ mice (Figures 2E,F). This study revealed that Panx1, which may be of neuronal or glial origin, participates in bidirectional synaptic plasticity by modulating both potentiation and depression in an age-dependent manner. Thus, the authors suggested that Panx1 channels may influence learning and memory in adults by restraining the threshold for the induction of synaptic plasticity. The functional significance of this age-dependent bidirectional regulation, however, is yet to be explored.

### Vision

**Figure 4:**
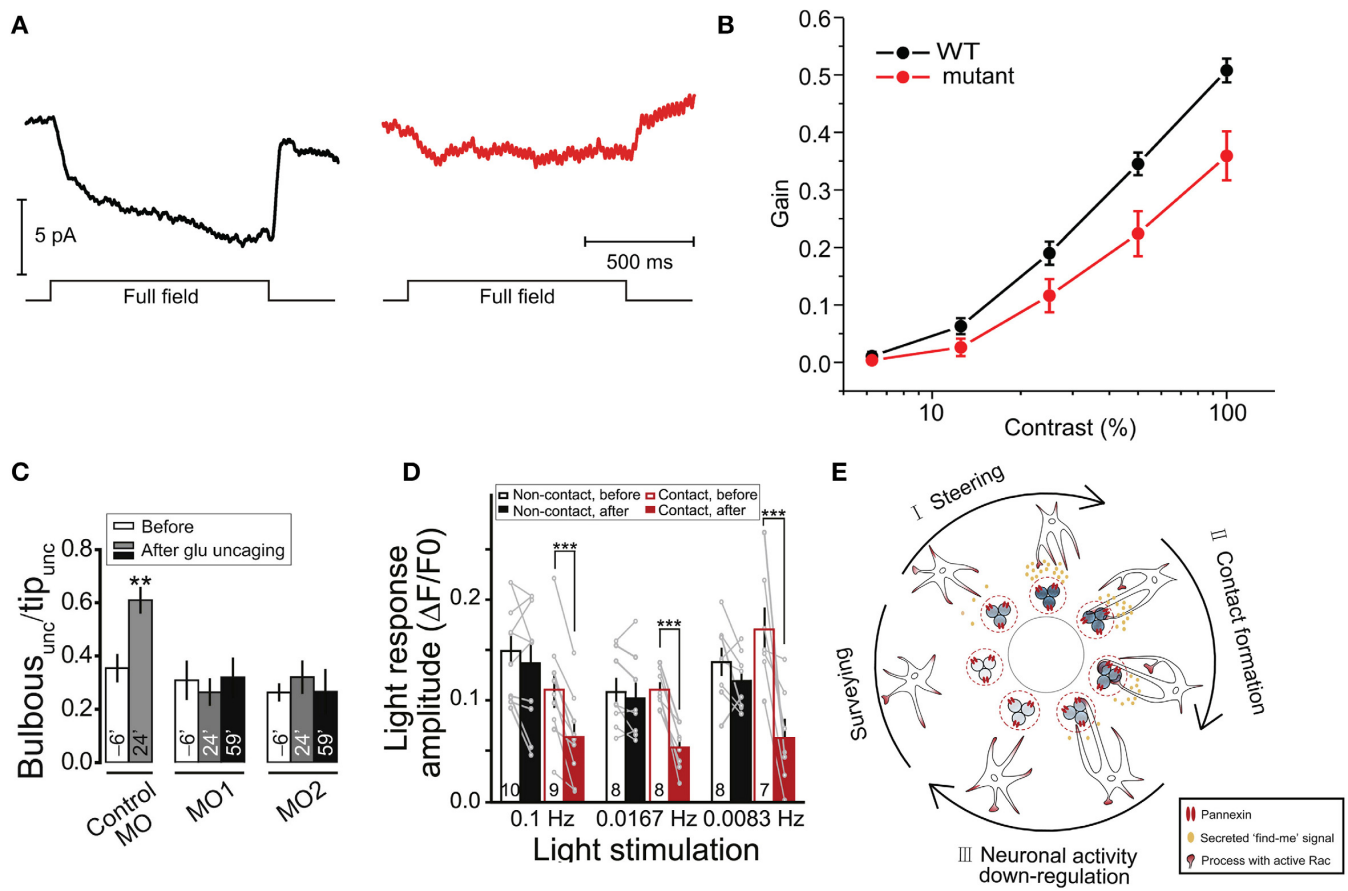
**Cx HCs and Panx1 channels have significant roles in synaptic transmission essential for vision. (A,B)** Cx55.5 HCs are important for contrast sensitivity in zebrafish retina. **(A)** To measure light-induced feedback responses, cones were first saturated with a 20 μm spot of light. A full-field light flash induced an inward current in cones due to negative feedback from horizontal cells. Cx55.5 mutant (red) cones showed a decreased feedback response compared to wild-type (black), as shown in sample traces. **(B)** Optokinetic gain, as a measure of contrast sensitivity, was determined by dividing the eye movement velocity by the velocity of the stimulus over a range of contrast in zebrafish larvae. This was significantly decreased in mutant compared to wild-type zebrafish. **(C–E)** Reciprocal regulation between resting microglia and neuronal activity via Panx1 channels. **(C)** Glutamate uncaging was performed in the intact zebrafish larvae to evoke Ca^2+^ activities of tectal neurons within 20 μm around the uncaging point of 1 μm in the soma layer of the optic tectum. From the side of microglia facing the uncaging point (“unc”), the proportion of the number of bulbous normalized to all process tips (“Bulbous_unc_/Tip_unc_”) is shown for larvae injected with splice morpholino oligonucleotides (MO) 6-min before (clear) and 24-min (gray) and 59-min (black) after uncaging. The increased in bulbous endings is shown in control MO, but abolished in Panx1 expression downregulation MO1 and MO2. **(D)** Normalized intensities of Ca^2+^ activities (light response amplitude) of tectal neurons *in vivo* evoked by moving bars at indicated frequencies are shown. Response is significantly reduced in neurons after microglial contact (red filled vs. clear bars) as compared to non-contact (black filled vs. clear bars). Numbers of neurons examined are shown on bars. **(E)** Schematic diagram showing a proposed model of microglial modulations of neuronal activity via Panx1 channels. During neuronal activity, neurons secrete “find me” signal locally (ATP being a candidate) via Panx1 channels, which steer microglial processes toward them (from “Surveying” to “I”). Bulbous endings are then formed on these processes promoting contact with neurons (“II”). Upon such contact, neuronal activity is downregulated (“III”). ^**^p < 0.01; ^***^p < 0.001. Adapted, with permission, from Klaassen et al. (2011) **(A,B)**, Li et al. (2012) **(C,D)**.

In addition to their contributions to synaptic and higher level brain functions, some fundamental visual processes have also been shown to be mediated by Cx HCs and Panx channels. In particular, two groups have demonstrated the involvement of these channels in visual processes in zebrafish. Between the two channels, Cx HCs were first implicated in synaptic transmission essential for vision (Kamermans and Fahrenfort, 2004; Klaassen et al., 2011). In the retina, Cx52.6 and Cx55.5 form GJ channels between horizontal cells, while Cx55.5 also form HCs at the tips of the horizontal cell dendrites and can open at physiological membrane potentials (Shields et al., 2007). Klaassen et al. (2011) generated zebrafish Cx55.5 mutant, with a stop codon in the first extracellular loop of Cx55.5 (C54X), which led to decrease in both, Cx55.5 expression on the tips of the horizontal cell dendrites, and HC currents from horizontal cells. They reported that the total gap-junctional surface was preserved, although alterations in Cx52.6 expression and in the general organization of gap-junctional plaques were found. This study investigated the negative feedback from horizontal cells to cones, essential for contrast enhancement in vision. In these mutants, light-induced feedback from horizontal cells to cones was significantly reduced (Figure 4A). As a consequence, these mutant zebrafishes exhibited impairment in contrast sensitivity (Figure 4B). Supported by a mathematical model, the authors suggested that current flowing through Cx HCs at the tips of horizontal cell dendrites actually exert an ephaptic modulation of synaptic transmission through activation of adjacent voltage-dependent Ca^2+^ channels in presynaptic photoreceptor terminals. Interestingly, they also reported an upregulation of Panx1 expression at the tips of the horizontal cell dendrites in these mutants, suggesting that it could be partly accountable for the residual 40% HC activity in the mutant. The same group later demonstrated that Cx55.5 HC currents possess an inward component that is active at physiological membrane potentials and extracellular Ca^2+^ levels (Sun et al., 2012). These studies suggest an unconventional role of horizontal cell Cx HCs in synaptic transmission in the retina, where a carp ortholog of mammalian Cx43 is also expressed among several other Cxs (Dermietzel et al., 2000). While similar mechanisms involving other Cxs and other CNS regions are yet to be investigated, these research findings further confirmed the dynamic nature of physiological roles of HCs in synaptic transmission leading to functional outcome, which in this case is visual acuity.

Another study revealed that neuronal activity in the optic tectum of larval zebrafish could modulate motility of resting microglia toward active neurons via Panx1 channels (Li et al., 2012). This in turn downregulates both spontaneous and visually-evoked neuronal activities under physiological condition. Specifically, using *in vivo* imaging techniques and local glutamate uncaging, the authors observed an enhanced formation of microglial processes with bulbous endings, which contact active neurons (Figure 4C). This was blocked by probenecid and CBX, as well as by injection of oligonucleotides which downregulate Panx1 expression, suggesting a potential contribution of Panx1 channel activity. As Panx1-mediated currents were recorded in tectal neurons but not in microglia, they concluded that this was due to the opening of neuronal Panx1 channels. The possible downstream involvement of ATP and P2 receptors was also tested. As a result, similar abolishment of activity-induced microglial bulbous ending formation was found in the presence of apyrase, an ATP-hydrolyzing enzyme, and suramin, a P2 receptor blocker. Remarkably, while microglia was found to preferentially contact neurons with initially high levels of spontaneous activity, the authors discovered that this spontaneous activity was reduced upon microglia-neuron contact. In addition, moving bar-evoked Ca^2+^ activity in these neurons was also decreased upon microglial contact (Figure 4D). These results suggest an interesting reciprocal modulation between neurons and microglia via neuronal Panx1 channels and ATP/P2 receptors (Figure 4E). The involvement of ATP as a “find-me” signal was expected, as activated microglia can be attracted toward sites of brain injury by ATP (Davalos et al., 2005). However, direct evidence of ATP release via Panx1 is yet to be shown. The authors postulated that since microglial processes mainly contact the soma, but not dendrites, of tectal neurons, such contact-induced downregulation is due to reduction of neuronal excitability rather than synaptic inputs.

## Conclusions, remaining questions and future directions

Unlike in pathology, the functional roles of Cx HCs and Panxs in physiological situations have only recently been considered and explored. This review summarizes the latest studies supporting their neurophysiological relevance in the CNS. In particular, aspects concerning development, glucose sensing, synaptic transmission and plasticity, learning and memory, as well as vision are discussed (Table 2). One of the first studies showed that ATP is released via Cx43 HCs from retinal pigment epithelial cells, which promotes division and proliferation of progenitor cells in the developing retina (Pearson et al., 2005). In the hypothalamic tanycytes, the same HC subtype contributes to the uptake of extracellular glucose, triggering downstream Ca^2+^ response via ATP release (Orellana et al., 2012a). Moreover, ATP released via Cx43 HCs in astrocytes was found to increase hippocampal excitatory transmission via P2 receptors (Chever et al., 2014). Upon decrease in extracellular Ca^2+^ levels, however, ATP released from astrocytic Cx43 HCs may also participate in the generation of feedback inhibitory transmission in hippocampal interneurons (Torres et al., 2012). Functionally, these channels have also been shown to play a role in fear memory consolidation in adult rats (Stehberg et al., 2012). Apart from Cx43, Cx55.5 HCs located at the tips of retinal horizontal cell dendrites were also found to conduct light-induced feedback transmission between horizontal cells and cones, and as a functional consequence, enhance contrast sensitivity of the eye (Klaassen et al., 2011). Similar to Cx43 HCs, ATP release from Panx1 channels was found to promote neurogenesis in the postnatal mouse ventricular zone (Wicki-Stordeur et al., 2012), as well as trigger a dowregulation of neuronal excitability upon decrease in extracellular glucose in the hippocampus (Kawamura et al., 2010). Interestingly, also in the hippocampus, they reduce synaptic transmission and exert an age-dependent bidirectional control (decreasing LTP while increasing LTD) over synaptic plasticity (Prochnow et al., 2012; Ardiles et al., 2014). In the zebrafish, Panx1 channels were found to be involved in promoting microglial motility toward active neurons, leading to a decrease in neuronal activity related to vision (Li et al., 2012). Lastly, they have also been shown to be important for sensorimotor gating, as well as object recognition and spatial memory (Prochnow et al., 2012). Taken together, these important studies suggest that Cx HCs and Panxs are not only open during physiological conditions, but also play important and dynamic roles in a variety of neurophysiological processes and behavior. With these encouraging findings, it is expected that more future evidence will emerge to strengthen the physiological relevance of these large pore membrane channels in the CNS. However, in order to achieve this, some important issues need to be considered and addressed.

**Table 2:** **Roles of connexin hemichannels and pannexin channels in neurophysiology**.

**Function**	**Channel subtype**	**Proposed mechanisms**	**Experimental models and tools**	**References**
**DEVELOPMENT AND GLUCOSE SENSING**				
**(1) Development**				
↑ Cell division and proliferation	Cx43	ATP released from RPE cells via Cx43 HCs triggers Ca^2+^ waves through RPE cells and stimulates P2 receptors on retinal progenitor cells	Isolated neural retina from chick embryos	Pearson et al., 2005
			*Pharmacological blocker (Carbenoxolone); mimetic peptide (Gap26)*	
	Panx1	ATP released via Panx1 channels stimulates NSC/NPC via P2 receptors	Neuro2a neuroblastoma cell line, cultures of postnatal mouse ventricular zone NSC/NPCs	Wicki-Stordeur et al., 2012
			*Pharmacological blocker (Probenecid); Panx1 siRNA*	
**(2) Glucose sensing and signal transduction**				
↑ Hypothalamic glucose-sensing	Cx43	Glucose uptake and glycolysis opens Cx43 HCs allowing the release of ATP, which activates local P2Y1 receptors and [Ca^2+^]_i_ increase	Cultured rat hypothalamic tanycytes	Orellana et al., 2012a
			*Pharmacological blockers (La^3+^, Cx43^E2^, Probenecid); mimetic peptides (Gap26, ^10^panx)*	
↑ Metabolic autocrine regulation	Panx1	Decrease in extracellular glucose triggers the opening of Panx1 channels through which ATP is released	Acute hippocampal slices from juvenile rats	Kawamura et al., 2010
			*Pharmacological blocker (Carbenoxolone, Octanol); mimetic peptide (^10^panx)*	
**SYNAPTIC TRANSMISSION AND PLASTICITY**				
**(1) Neuronal excitability and synaptic transmission**				
↑ Synaptic transmission	Cx43	ATP released via Cx43 HCs in astrocytes promotes excitatory synaptic transmission via P2 receptors	Acute hippocampal slices from juvenile mice	Chever et al., 2014
			*Transgenic mice: Cx43^fl/fl^:hGFAP-Cre = Astrocytic Cx43 conditional knockout; pharmacological blocker (Carbenoxolone), mimetic peptide (Gap26, ^10^panx)*	
↑ Feedback inhibitory synaptic transmission	Cx43	Decrease in [Ca^2+^]_e_ during excitatory transmission opens Cx43 HCs in astrocytes allowing the release of ATP, which triggers depolarization and firing of inhibitory interneurons and a slow Ca^2+^ wave in astrocytes both via P2Y receptors	Acute hippocampal slices from juvenile mice	Torres et al., 2012
			*Transgenic mice: (1) Cx30^−/−^Cx43^*fl/fl*^:hGFAP-Cre = Cx30 total- and Cx43 astrocytic conditional knockout; (2) Cx30^−/−^Cx43^*fl/fl*^ = Cx30 total knockout; and 3) Cx43^+^/*G138Rfl*:hGFAP-Cre mice = enhanced astrocytic Cx43 HC activity; pharmacological blocker (Carbenoxolone)*	
↓ Neuronal excitability	Panx1	Decrease in extracellular glucose triggers the opening of Panx1 channels through which ATP is released. Its metabolite adenosine then activates adenosine A1 receptors and opens K_ATP_ channels leading to decreased neuronal excitability	Acute hippocampal slices from juvenile rats	Kawamura et al., 2010
			*Pharmacological blocker (Carbenoxolone, Octanol); mimetic peptide (^10^panx)*	
↓ Synaptic transmission	Panx1	Adenosine metabolized from ATP released via Panx1 channels decreases neurotransmission	Acute hippocampal slices from adult mice	Prochnow et al., 2012
			*Transgenic mice: Panx1^−/−^ (Panx1 knockout)*	
	Panx1	Panx1 channels decrease neurotransmission in adult but not young mice	Acute hippocampal slices from young and adult mice	Ardiles et al., 2014
			*Transgenic mice: Panx1^−/−^ (Panx1 knockout); Pharmacological blocker (Probenecid)*	
↑ Light-induced feedback transmission	Cx55.5	Cx55.5 HC current at the tips of the horizontal cell dendrites induces local voltage drop near voltage dependent Ca^2+^ channels of cones mediating feedback ephaptic transmission	Isolated retina, dissociated horizontal cells and cones from zebrafish	Klaassen et al., 2011
			*Transgenic zebrafish: Cx55.5 mutant (no functional Cx55.5 protein formation)*	
↓ Spontaneous neuronal activity	Panx1	Panx1 channels on active neurons promote microglial motility via ATP/P2 receptors, which in turn downregulates spontaneous neuronal activity	Zebrafish larvae	Li et al., 2012
			*Pharmacological blocker (Carbenoxolone, Probenecid); Panx1 downregulation oligonucleotides*	
**(2) Synaptic plasticity**				
↓ LTP	Panx1	Adenosine metabolized from ATP released via Panx1 channels suppresses the induction of LTP	Acute hippocampal slices from adult mice	Prochnow et al., 2012
			*Transgenic mice: Panx1^−/−^ (Panx1 knockout); Pharmacological blocker (mefloquine)*	
↓ LTP, ↑ LTD	Panx1	Panx1 channels control the threshold of the bidirectional induction of synaptic plasticity in adult but not in young mice	Acute hippocampal slices from young and adult mice	Ardiles et al., 2014
			*Transgenic mice: Panx1^−/−^ (Panx1 knockout); Pharmacological blocker (Probenecid)*	
**INTEGRATIVE FUNCTIONS**				
**(1) Sensorimotor gating**				
↓ Sensorimotor gating capabilities	Panx1	Panx1 channels inhibit pre-pulse inhibition of the acoustic startle response	Mice (adult)	Prochnow et al., 2012
			*Transgenic mice: Panx1^−/−^ (Panx1 knockout)*	
**(2) Learning and memory**				
↑ Fear memory consolidation	Cx43	Gliotransmitters release through astrocytic Cx43 HCs promotes the consolidation of fear memory	Rats (adult)	Stehberg et al., 2012
			*Mimetic peptides injection into basolateral amygdala (Gap26, TAT-Cx43L2)*	
↑ Object recognition memory	Panx1	Panx1 channels improve the ability of a mouse to recognize an object as familiar	Mice (adult)	Prochnow et al., 2012
			*Transgenic mice: Panx1^−/−^ (Panx1 knockout)*	
↑ Spatial memory	Panx1	Panx1 channels enhance the ability of a mouse to remember the location of a treat	Mice (adult)	Prochnow et al., 2012
			*Transgenic mice: Panx1^−/−^ (Panx1 knockout)*	
**(3) Vision**				
↑ Contrast sensitivity	Cx55.5	Cx55.5 HC mediated feedback from horizontal cells to cones leads to contrast enhancement	Zebrafish larvae	Klaassen et al., 2011
			*Transgenic zebrafish: Cx55.5 mutant zebrafish (no functional Cx55.5 protein formation)*	
↓ Visually-evoked neuronal activity	Panx1	Microglial contact promoted by Panx1 channels on active neurons via ATP/P2 receptors downregulates evoked neuronal activity	Zebrafish larvae	Li et al., 2012
			*Pharmacological blocker (Carbenoxolone, Probenecid); Panx1 downregulation oligonucleotides*	

Cx, Connexin; fl, floxed; HC, hemichannel; G138R, single point mutation of glycine 138 to arginine of Cx43; LTD, long-term depression; LTP, long-term potentiation; NSC/NPS, neural stem cells and progenitor cells; Panx, Pannexin; RPE, retinal pigment epithelium.

### What are the composition of functional HCs and substances released?

Current understanding of the physiological roles of Cx HCs and Panx channels is largely focused on two specific subtypes, namely Cx43 and Panx1, both permeable to ATP. The lack of focus on other channel isoforms however does not rule out their potential contributions in physiology. Although Torres et al. (2012) have shown that Cx30 does not account for the hippocampal defects that they observed in Cx30/Cx43 double knockout mice, it is unclear whether Cx30 has any HC-specific effect on neurotransmission. Indeed, one major difficulty in the study of HCs is the lack of experimental tools with high isoform selectivity, as well as specificity for HCs over GJ channels. For instance, the pharmacological blocker CBX acts on Cx HCs and Panx channels, as well as on GJs (Schalper et al., 2008). Similarly, Gap26/27, which are mimetic peptides commonly used to block Cx43 HCs, can also affect GJ channels when applied for several hours (Samoilova et al., 2008). Nevertheless, a few inhibitors that block HCs but not GJs, such as Gap19/TAT-Gap19 and TAT-L2 (see Table 1), have been developed (Ponsaerts et al., 2010; Wang et al., 2013b). Therefore, advances in the generation of more specific mimetic peptides and even antibodies (Riquelme et al., 2013) should improve selectivity among HC isoforms. Transgenic organisms with impairment in either gap-junctional or HC functions would also be ideal. Furthermore, it is interesting that most of the studies described in this review have demonstrated effects mediated by ATP release or its metabolite, adenosine. As HCs are also permeable to signaling molecules like NAD^+^, glutamate, glutathione and prostaglandin E2 (Wang et al., 2013a), which are known to be important for synaptic activity and cognitive functions, it is of interest to expand our search toward the effect of the release of these substances.

### Where are functional HCs located?

The fact that physiologically active HCs are detected in a number of cell types ranging from stem cells to neurons and glia implies their ubiquitous functions in the CNS (see Table 2). In fact, Cx43 proteins are largely expressed in vessels (Simard et al., 2003; Rouach et al., 2008) and supposedly near synapses (Ormel and Gundersen, 2011), suggesting their roles as regulators of synaptic activity, vasomotricity and hyperemia. In addition, their preferential expression on astrocytes facilitates their roles in astroglial network excitability, mediating paracrine (Torres et al., 2012) and autocrine regulations within astrocyte network (Wang et al., 2013a). Some neurons also express HC proteins like Cx36 in GABAergic interneurons or Panxs in pyramidal neurons, but their properties as HCs *in vivo* is still under investigation. Furthermore, both Cx HCs and Panx1 channels have also been found to be expressed on microglia and oligodendrocytes with activities associated with pathological ATP and glutamate release, as well as ion gradient imbalance (Orellana et al., 2011c). Their functions in physiology, however, are yet to be explored.

Other than cell-type specific expression, it is also unclear in which subcellular domain functional HCs are present. For instance, Cx43 HCs expressed on astroglial plasma membranes have been thought to serve as a reserve pool for GJ formation. Thus, it has been a challenge to differentiate between functional HCs and those presented on the membrane for GJ activities using only immunostaining. The development of new techniques and tools to not only study, but to also visualize the location of active HCs, is therefore warranted. We have recently shown that astrocytes can extend their fine processes near or even within synaptic clefts (Pannasch et al., 2014), suggesting that the expression of functional HCs on these processes would allow them to more efficiently modulate neuronal transmission due to their proximity to targeted sites. Finally, if HCs are concentrated on subcellular domains, it would be interesting to investigate how they are targeted and trafficked to these locations.

### When are HCs open?

While numerous *in vitro* studies have been performed to characterize the biophysical properties of these large membrane pores and to determine aspects that would trigger their opening, HC activation *in situ* or *in vivo*, particularly during physiological conditions, is less understood. Torres et al. (2012) proposed that the increase in glutamate and decrease in extracellular Ca^2+^ occurring during synaptic transmission favor astroglial Cx43 HC opening, suggesting that the conductance or open probability or number of active channels, or possibly recruitment of HCs near synaptic cleft, is dependent on neuronal activity. Although they also used high frequency stimulation to mimic neuronal activity, it is not clear as to whether these manipulations represent conditions *in vivo* and if the suggested mechanism is involved in the modulation of cognitive functions (Stehberg et al., 2012). Apart from extracellular Ca^2+^, an increase in intracellular Ca^2+^ within the physiological range has also been found to open Cx43 and Cx32 HCs, as assessed by ATP release and dye uptake (De Vuyst et al., 2006, 2009). On the other hand, studies on Panx1 channels have reported cognitive and behavioral deficits in knockout mice (Prochnow et al., 2012). However, long-term effect due to chronic deletion of the gene cannot be ruled out. The development in the application of current techniques *in vivo*, especially those that allow acute manipulation or detection of channel opening and closing, would be useful for addressing these issues. More importantly, the use of such *in vivo* systems will offer the benefit of behavioral studies, cognitive tests as well as the characterization of developmental defects.

It is conceivable that, due to their large pore size and conductance, the opening of Cx HCs and Panx channels is different from other plasma membrane ion channels and likely requires tighter control to preserve cell integrity. This more stringent regulation could be related to minimizing channel opening duration. Perhaps various modulators could differentially open the same channel with different timescales. Transient channel opening to release substances in short pulses may also prevent a sudden loss of intracellular substances. In contrast, Cx HC and Panx channels opening might merely be a passive response toward transient changes in concentration gradients of molecules like glucose, lactate, or ATP in an activity-dependent manner contributing to the maintenance of basal homeostasis. Additionally, HCs are permeable to water and could play an important role in regulation of intracellular osmolarity and cell volume (Quist et al., 2000). To test these possibilities, it becomes essential to focus on the moment-to-moment action of HC activity on brain processes like neurotransmission.

### How are HC opening and substance release regulated for downstream effects?

HCs are rather non-selective large pore membrane channels which, if not regulated, could lead to loss of important intracellular molecules and more seriously cell death. Thus, the open probability of these channels, their membrane distribution, trafficking and proximity to target receptors are crucial aspects requiring tight controls. While the opening and closing of Cx GJ channels are tightly regulated (Dermietzel and Spray, 1993; Pannasch and Rouach, 2013), it is not well understood whether the same pathways also apply to HCs. More importantly, it is still unclear which molecular mechanisms underlie the formation of HCs over GJ channels and vice versa. It has been shown in pathological situations that GJ channels and HCs can display similar or opposite responses depending on differential activation of intracellular pathways. For example, lipopolysaccharide and basic fibroblast growth factor have been found to differentially influence Cx HCs and GJ channels (De Vuyst et al., 2007). In relation to this, aspects of how they are targeted to and maintained on plasma membranes as single channels are also fundamental to understand physiological HC properties.

Interestingly, Cx43 HCs and Panx1 channels seem to have opposite roles on neuronal activity and behavior. While Stehberg et al. (2012) showed that inhibiting Cx43 HCs with mimetic peptides *in vivo* strongly impaired fear memory consolidation, two other subsequent studies on Panx1^−/−^ mice demonstrated a strong enhancement in LTP and an abolishment in LTD (Prochnow et al., 2012; Ardiles et al., 2014). This suggests that Cx43 and Panx1 HCs are involved in two distinct neuromodulatory pathways. One can also argue that such differential effect might rely on differences in experimental models using acute blockage of Cx43 HC as opposed to Panx1^−/−^ mice with chronic deletion of channels. Indeed, the effects on LTP and LTD were not observed in young mice, but only in adults, in which they also observed a compensatory transcriptional up-regulation of metabotropic glutamate receptor 4, proposed to be due to chronic decrease in extracellular ATP. Furthermore, Stehberg et al. only showed that the release via Cx43 HCs involved one or more gliotransmitters, whereas Panx1^−/−^ defects were specifically rescued by adenosine. This raises the questions of whether these HCs could exhibit cell-type specific regulations (in this case neurons vs. astrocytes) or even release different combinations of molecules under varying circumstances. In addition, the quantity of release and the proximity to targeted sites, as well as the nature and localization of receptors, also need to be taken into account. It is of great importance that such opposing effects between HC subtypes are clarified such that any differential modulations of downstream effects can be better understood. The use of global and conditional knockout systems will provide valuable insights into whether the observed effects are cell-type or developmentally specific. It would also be important to determine whether HC uptake and release of various transmitters, ions and metabolites are co-regulated and whether it is synergistic with other release pathways. This may enhance the efficiency of cell-to-cell communication and allow dynamic actions on downstream mechanisms.

On a more complex level, it would be of great interest to determine if HC activity could display plasticity similar to postsynaptic glutamate receptors. It is probable that in response to neuronal activity, HC conductance or phosphorylation is altered leading to differential expression/presentation and distribution accross plasma membrane. Indeed, several Cx HCs, like Cx43 and Cx46, have been shown to have increased open probability depending on their phosphorylation state (Li et al., 1996; Ngezahayo et al., 1998; Sáez et al., 2005). Lastly, while most physiological actions of HCs thus far involve ATP release, it is necessary to clarify whether different channels indeed have specific and/or exclusive roles in certain biophysical processes contributing to neurotransmission and behavior. If so, what underlying mechanisms would allow them to be differentially regulated. As ATP is an important substance regulating neurotransmission and many basal processes, similar roles of HCs on other functions like hyperemia (Pelligrino et al., 2011) and brain oscillations (Schulz et al., 2012) are likely and yet to be characterized.

While there has been decades of research to establish the pathological contributions of HCs in the CNS, our understanding of their potential roles in physiological processes has just begun. Indeed, as more encouraging findings emerge, many fundamental issues must be addressed. Nevertheless, it is hoped that by unraveling the properties of these membrane channels in physiology, we will also be able to gain insights into their functions in pathologies and to explore their potential roles as therapeutic targets.

### Conflict of interest statement

The authors declare that the research was conducted in the absence of any commercial or financial relationships that could be construed as a potential conflict of interest.
